# Physico-mechanical and morphological features of zirconia substituted hydroxyapatite nano crystals

**DOI:** 10.1038/srep43202

**Published:** 2017-03-03

**Authors:** S. F. Mansour, S. I. El-dek, M. K. Ahmed

**Affiliations:** 1 Physics Department, Faculty of Science, Zagazig University, Egypt; 2 Materials science and nanotechnology Department, Faculty of Postgraduate Studies for Advanced Sciences (PSAS), Beni-Suef University, Egypt; 3 Materials Science Lab.(1), Physics Department, Faculty of Science, Cairo University, Giza, Egypt

## Abstract

Zirconia doped Hydroxyapatite (HAP) nanocrystals [Ca_10_(PO_4_)_6−x_(ZrO_2_)_x_(OH)_2_]; (0 ≤ x ≤ 1 step 0.2) were synthesized using simple low cost facile method. The crystalline phases were examined by X-ray diffraction (XRD). The crystallinity percentage decreased with increasing zirconia content for the as-synthesized samples. The existence of zirconia as secondary phase on the grain boundaries; as observed from scanning electron micrographs (FESEM); resulted in negative values of microstrain. The crystallite size was computed and the results showed that it increased with increasing annealing temperature. Thermo-gravimetric analysis (TGA) assured the thermal stability of the nano crystals over the temperature from room up to 1200 °C depending on the zirconia content. The corrosion rate was found to decrease around 25 times with increasing zirconia content from x = 0.0 to 1.0. Microhardness displayed both compositional and temperature dependence. For the sample (x = 0.6), annealed at 1200 °C, the former increased up to 1.2 times its original value (x = 0.0).

Bone is a biological composite of two components; collagen; the biological part, and hydroxyapatite (HAP) which is composed of rod like particles, similar to the mineral part embedded in the collagen fibrils^1^,^2^. Bone’s apatite is not a pure HAP; it is naturally doped with different elements and concentrations^3^. Synthesized HAP is distinguished by its good biocompatibility, osteoconductivity and bone-attachment ability but its brittleness, rigidity and high degradation rate have limited HAP from being used in the broad spectrum of biomedical applications^4^,^5^,^6^,^7^.

Several researchers synthesized HAP; focusing on the development of powder processing methods, composition adjustment and preparation conditions, to find the most effective preparation technique which achieves high strength HAP without altering the biocompatibility nature^5^. One of the reported works was the synthesis of HAP with large surface area. The results^8^ displayed sinterability and enhanced densification which contributes to the improvement of the mechanical properties.

Ionic incorporations in HAP ceramics, such as silver (Ag), magnesium (Mg), iron (Fe), copper (Cu), strontium (Sr) and zinc (Zn), have gained more interest due to their role in the biological processes after implantation^9^. Most of these ionic substitutions were incorporated into Ca^2+^ position. The (PO_4_)^3−^ could also be substituted by (CO_3_)^2−^ to produce B-type carbonated HAP. Other researchers mentioned that the later can be replaced by silicate^10^ (SiO_4_)^4−^.

S. H. An *et al*. fabricated ZrO_2_/HAP scaffolds and they get good mechanical properties with higher cell affinity with no detectable degradation^11^. K. Castkova *et al*. synthesized microporous zirconia/HAP composites. Due to the diffusion of Ca^2+^ from HAP to ZrO_2_, HAP transformed into a mixture of HAP, α and β-tri calcium phosphate (TCP) and calcium zirconate. In addition, Young’s modulus and hardness of the composite depended on the internal porosity level^12^. The addition of zirconia caused a decrease in HAP grain size in the porous HAP as investigated by C. Yi Chiu *et al*.^13^.

Z. Evis studied the reactions in zirconia/HAP composites. He concluded that the HAP unit cell volume increased with increasing zirconia content due to the ionic exchange of Ca^2+^ and ZrO^2+^ between HAP and zirconia^14^. This ionic exchange was found to increase the ionic conductivity as concluded by M. Inuzuka *et al*.^15^. The phase stability of zirconia/HAP was examined by A. R. Kmita *et al*. They reported that the transformation of ZrO_2_ from monoclinic to tetragonal phase was responsible for the reinforcing mechanism^16^. The bending strength of HAP was improved more than 25% when 1.5 wt % of tetragonal zirconia was added^17^. Additionally, the bending strength and the bulk density of zirconia/HAP increased with annealing temperature in composites synthesized by K. Yoshida *et al*.^18^. No detectable reaction between zirconia and HAP phases annealed at 1450 °C was reported by J. Zhang *et al*.^19^. Furthermore, the reduction in strength was probably related to the residual porosity in zirconia/HAP by Y. Nayak *et al*.^20^. Z. E. Erkmen *et al*.^21^ correlated microstructural and mechanical properties of zirconia/HAP. Summarizing their results; the hardness behavior supported the density data. Besides, the decomposition of HAP to β and α-TCP increased with rising zirconia concentration due to the interaction between CaO with zirconia^21^.

One of the most common applications is the use of HAP as a coating layer on zirconia (ZrO_2_) nano particles. This resulted in higher bonding strength, improved mechanical properties, biocompatibility and high corrosion resistance^22^. V. V. Silva *et al*. synthesized composite of partially stabilized zirconia/HAP. They discussed the hypothetical incorporation of Ca^2+^ by Zr^4+^ in the HAP structure. They supposed that the mechanism is that each 2 Ca^2+^ ions are replaced by one Zr^4+^ to keep the charge balance and symmetry^23^. N. Kawashima *et al*. investigated the surface characteristics of the annealed HAP/zirconia composite. They concluded that the HAP/zirconia has a mechanical compatibility as well as a potential for good biocompatibility with bone tissue^24^.

S. Salehi *et al*. synthesized HAP/zirconia composite. They investigated the crystalline structure and found that the presence of zirconia particles on HAP grain boundaries inhibits the grain growth of HAP^25^. T. J. Matsumotoa *et al*., studied HAP/zirconia composite with microporous structure and they found that the later exhibited high protein adsorption and encouraged cellular affinity. They argued that the investigated composite possesses excellent strength which is equivalent to that of cortical bones^26^.

J. B. Miecznik *et al*. have studied the addition of zirconia on HAP hot pressed materials and the results presented extraordinary enhancement of hardness and mechanical strength compared to pure HAP^27^. However, M. J. Lukić *et al*.have synthesized HAP nano powder in the presence of zirconium (Zr^4+^) ions, keeping (Ca + Zr)/P = 1.67. The authors reported that the phase transformations could be tailored by adjusting zirconium content^28^,^29^.

All reported literature was concerned with one of the following (i) coating zirconia surface by HAP nanoparticles, (ii) preparing (HAP + zirconia) as binary or ternary composite, (iii) substituting Ca^2+^ by zirconia in HAP matrix with different concentrations. However, the influence of zirconia incorporation in HAP matrix on (PO_4_)^3−^ crystallographic site have been scarcely reported. In this piece of work, we aimed to investigate the effect of zirconia doping content on HAP microstructure and explore the physical properties of the obtained nanocrystals.

## Results

### Crystal structure determination

**Figure 1:**
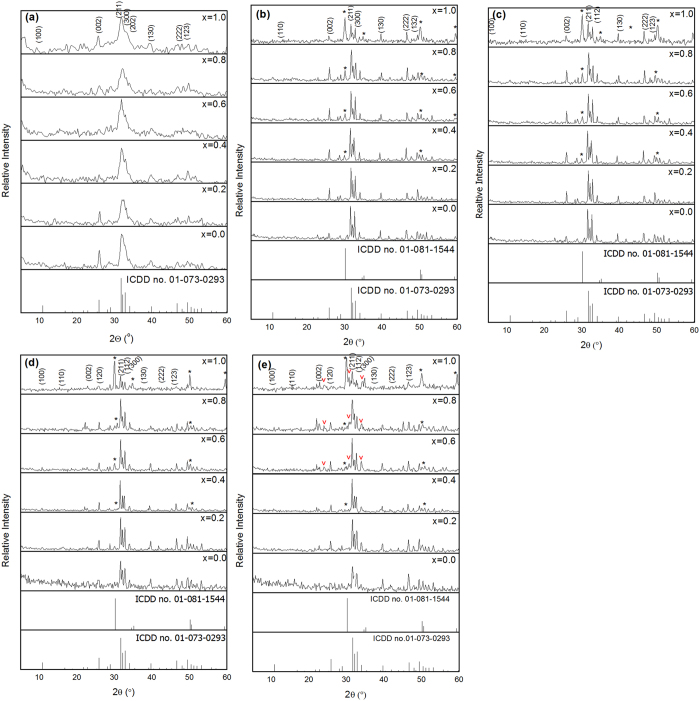
XRD with different zirconia content at different annealing temperatures, (**a**) as synthesized samples, (**b**) 1000 °C, (**c**) 1100 °C, (**d**) 1200 °C, (**e**) 1300 °C, (*: ZrO_1.95_, v: α-TCP).

XRD patterns of the as-synthesized HAP dopant with zirconia (Zr-HAP) samples are presented in Fig. 1(a). The obtained diffraction peaks matched well with those in the ICDD card no. (01-073-0293). As illustrated, there are no other foreign phases. XRD of the annealed samples (1000, 1100, 1200 and 1300 °C) are illustrated in Fig. 1(b–e), respectively. All HAP samples were crystallized in a hexagonal symmetry and indexed with ICDD card no. 01-073-0293. The lines appeared at 2θ = 30.177°, 50.182°, 60.029° are indexed as zirconia as compared to ICDD card no. 01-081-1544. The peak intensities increased as a zirconia content increased as well as with increasing the annealing temperature.

In case of the samples annealed at 1300 °C in Fig. 1(e), another diffraction peak appeared at x ≥ 0.6 and was attributed to α-TCP; as matched with the ICDD card no. 01-070-0364.

**Figure 2:**
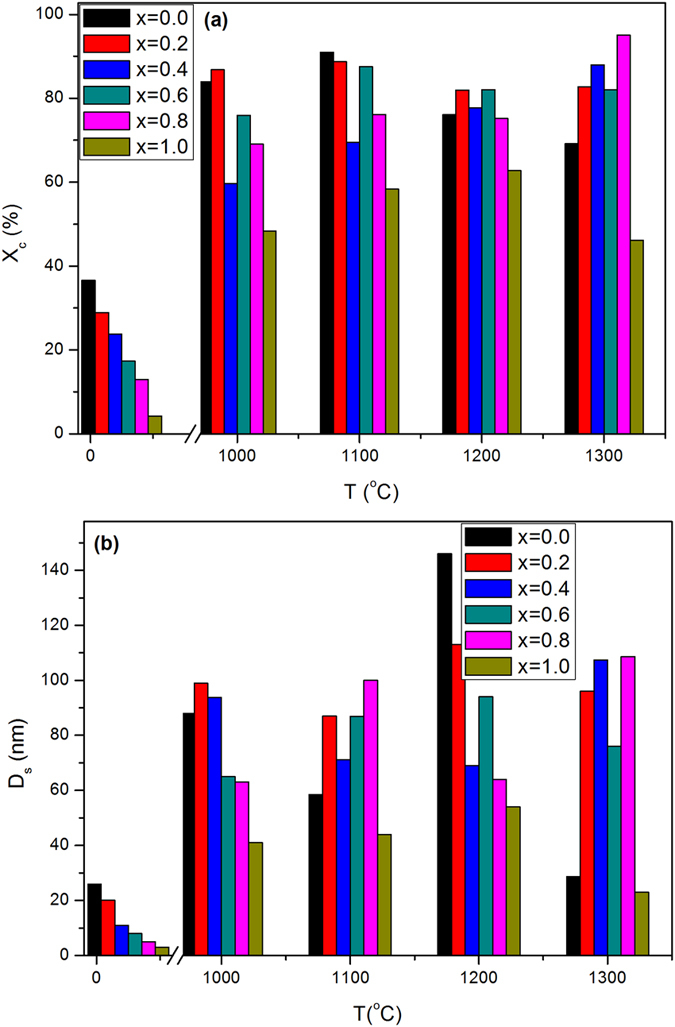
Values of crystallinity and crystal size (**a**) crystallinity of the as synthesized and annealed samples at different contents of zirconia, (**b**) crystallite size from Sherrer’s equation for the as-synthesized and annealed samples at different contents of zirconia.

The crystallinity was illustrated in Fig. 2(a) as a function of zirconia content and was lowered with increasing (ZrO_2_) content. There is a large difference in crystallinity between the as-synthesized and annealed samples. For the annealed samples, it varies from 46.2 up to 95.1%, however, it doesn’t exceed 36.6% in case of the as-synthesized samples.

The crystallite size was plotted versus x in Fig. 2(b), where it has a similar trend to the crystallinity. The former varies from 28.7 up to 120.1 nm at 1300 °C. The as-synthesized samples displayed maximum value of the crystallite size 26.2 nm at x = 1.0. For zirconia, the crystallite size was calculated separately at x = 1.0 at different annealing temperatures, where the former was found to increase with annealing temperature as reported in (Table 1).

**Figure 3:**
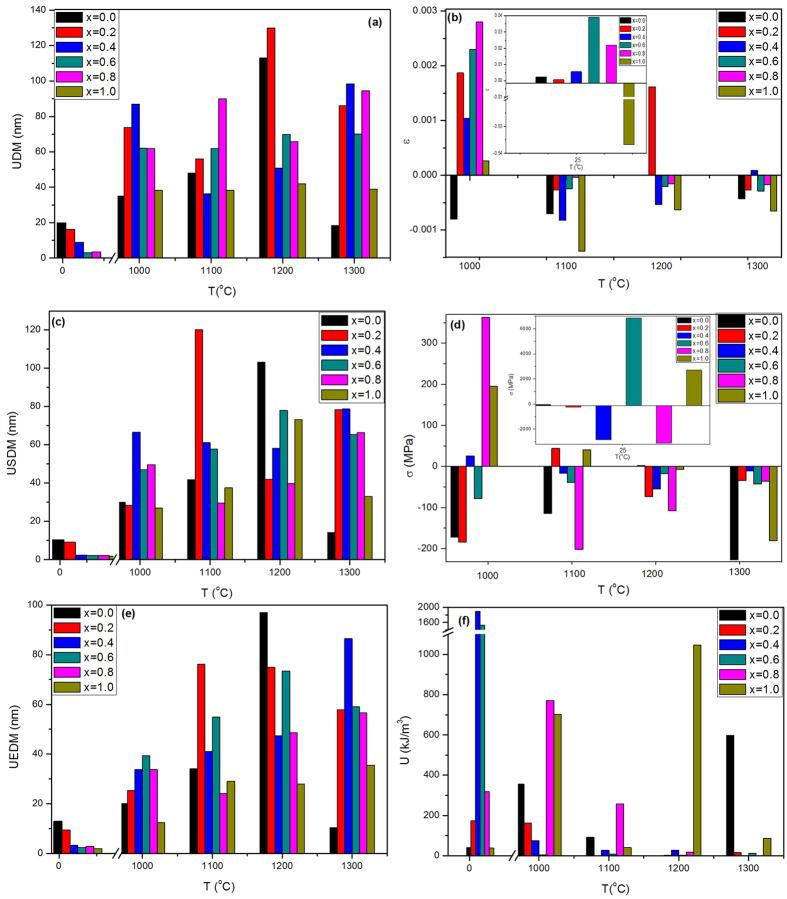
(**a**) Crystallite size calculated from UDM model, (**b**) lattice strain of the annealed samples and the inset plot for the as synthesized samples, (**c**) crystallite size calculated from USDM model, (**d**) stress of the annealed samples and the inset plot for the as synthesized one, (**e**) crystallite size calculated from UEDM model, (**f**) energy density of the annealed samples. All diagrams are plotted at different zirconia contents.

The microstrain (ε) and crystal size contributions, that are independent factors, result in the total broadening of the diffraction peaks^30^. Accordingly, the total peak broadening is represented by the sum of the impacts of crystallite size and strain existing in the sample. The uniform distribution model (UDM) assumes uniform strain in all crystallographic directions for all samples are illustrated in Fig. 3(a) as a function of zirconia content.

The UDM crystal size behavior is found to be decreasing with small values in most cases, while for the as-synthesized samples, UDM was slightly decreased. As reported in (Table 1); the UDM crystal size of zirconia at x = 1, increased with annealing temperature.

The lattice strain calculated from UDM, was plotted in Fig. 3(b) and reveals a trend opposite to that of crystal size where it increased with increasing x. The maximum of the curve was achieved at x = 0.8 for the annealed samples. The largest value was obtained for the as-synthesized sample and reached −536.78 × 10^−3^. The strain for the annealed samples varied from −4.29 × 10^−3^ up to 2.8 × 10^−3^. Zirconia phase possesses minimum lattice strain as compared with that calculated for HAP which varied from 0.35 × 10^−3^ to −2.78 × 10^−3^.

The relation between stress and strain is expressed by Hook’s law, which is valid for significantly small strains. As illustrated in Fig. 3(c), the uniform stress deformation model (USDM) crystal size displays increasing and decreasing behavior for the annealed samples. It increases and reaches maximum values at x = 0.2 to 0.6 then decreases slightly. The USDM crystal size behavior of the as-synthesized samples is similar to UDM one where the former decreases with x. The stress was calculated and plotted versus x at Fig. 3(d), which shows that their values for the as-synthesized samples are larger than those of the annealed samples.

Hook’s law assumed the homogeneous isotropic nature of the crystal. However, in real samples, the assumption of perfect isotropy is not fulfilled. Moreover, all the proportionality constants associated with the stress/strain relation are no longer independent when the strain energy density u is considered.

The uniform energy deformation mode (UEDM) crystal size varies in a decreasing manner for the annealed samples while for the as-synthesized samples, it decreases as shown in Fig. 3(e). The energy densities are shown in Fig. 3(f) and shows an opposite trend to that of the UDM lattice strain, where low values of the former were obtained from x = 0.2 to x = 0.6 and then increased significantly.

**Figure 4:**
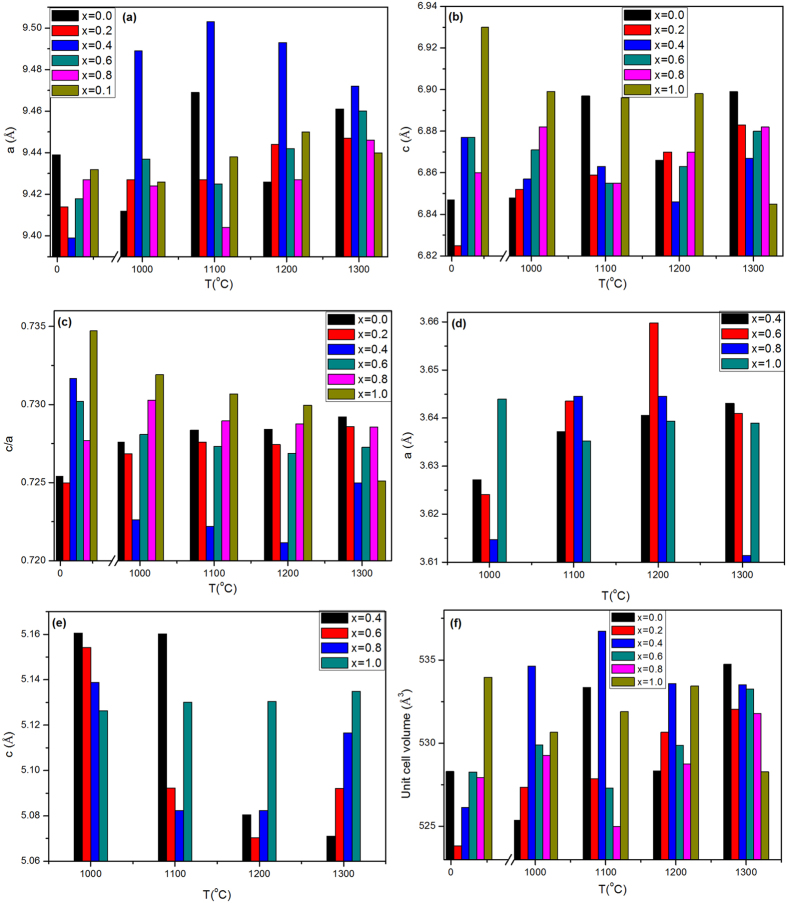
(**a**–**c**) Calculated lattice constant of hydroxyapatite (**a**) and (**c**) and (c/a) of the as synthesized and annealed samples at different zirconia content, respectively, (**d**,**e**) calculated lattice constant (**a** and **c**) of the zirconia unit cell at different annealed temperatures, (**f**) unit cell volume of hydroxyapatite unit cell for the as synthesized and annealed sample at a different content of zirconia.

From the crystallographic data illustrated in Fig. 4(a–f), the lattice constant (a) of the annealed samples increases with the zirconia content up to x = 0.4 then decreases in a Gaussian like distribution, with the highest value of a = 9.503 Å at 1100 °C. The samples at 1300 °C followed the same behavior with a smooth trend. The as-synthesized samples illustrated the opposite trend, where (a) reveals the minimum value at x = 0.4 as viewed in Fig. 4(a).

The (c) constant of the HAP unit cell increased slightly as shown in Fig. 4(b). In addition; the c/a ratio represents a reverse style to the (a) constant. It reaches the highest value at x = 0.4 for the as-synthesized and the least one at 1200 °C as Fig. 4(c). On the other side, the (c) parameter for zirconia decreases quickly in case of 1200 and 1300 °C. However, it possesses an opposite trend at 1000 and 1100 °C samples. It is noted that a and c constants of zirconia have a converse feature as illustrated in Fig. 4(d,e).

The HAP unit cell volume has the same trend of (a) constant, the largest unit cell volume is 536.739 Å^3^ at x = 0.4 as illustrated in Fig. 4(f).

**Figure 5:**
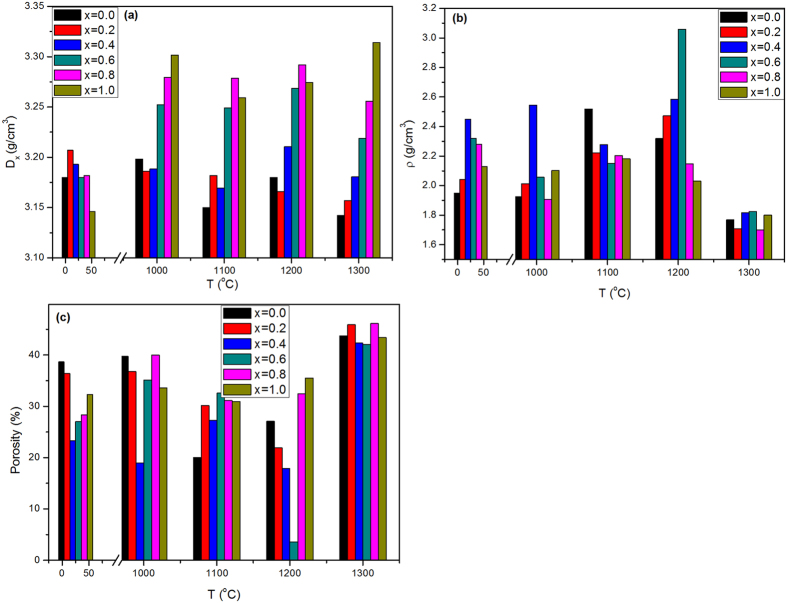
(**a**) Theoretical density, (**b**) measured density, (**c**) calculated porosity for the as synthesized and annealed samples with different contents of zirconia.

The theoretical density (D_x_) was determined using the formula^31^:


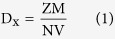


where M is the molecular weight of the sample, N is Avogadro’s number, Z is the number of molecules per unit cell and V is the unit cell volume. The modified theoretical density^12^:


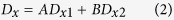


where A, B are the relative contribution ratios of the existing crystalline phases. As illustrated in Fig. 5(a), the theoretical density has significant increasing behavior with increasing the zirconia ratio in all annealed samples. However, it has an inverse trend in case of the as-synthesized samples.

The measured densities yield different trends. They increased considerably with x increasing up to x = 0.4, then decreased again as clarified in Fig. 5(b).

The porosity data; the ratio between the theoretical and measured density^32^; displayed the inverse trend of the measured density presented in Fig. 5(c).


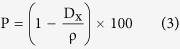


### FTIR

**Figure 6:**
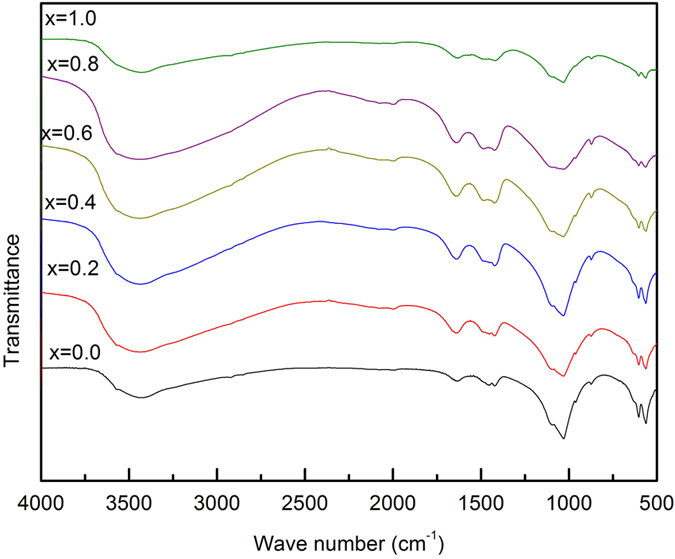
FTIR spectrum of the as synthesized samples at different contents of zirconia.

The FTIR spectrum of the as-synthesized Zr-HAP at different zirconia concentrations is shown in Fig. 6. The recognizable bands were detected as follows: At x = 0.0, the spectra shows distinct vibrational bands at 602.5 and 562.8 cm^−1^, attributed to the ν_4_ vibrational mode, while the weak intensity band at 467.9 cm^−1^ as a component of the ν_2_ mode corresponds to P-O bending^33^,^34^,^35^. The band at 525.1 cm^−1^ that appeared only at x = 1.0 is assigned for Zr–O stretching vibration mode^28^,^36^,^37^.

The bands appeared at 1453.7, 1421.8 and 874 cm^−1^ correspond to the (ν_3_) asymmetric stretching vibrations and bending (ν_2_) modes of carbonate groups^38^, respectively as appeared in HAP at x = 0.0. The position of the carbonate bands shows predominately B-type HAP, which is the favored substitution in human bones, known for its excellent bioactivity and osteoinductivity^33^. There is no A-type in all samples. However, at x = 0.0; the bands of 447.5 and 467.9 cm^−1^ are referred to ν_2_ (bending mode) of PO_4_^3−^ ions while those at 562.8 and 602.5 cm^−1^ are assigned to ν_4_ (bending mode) as well as 1030.3 cm^−1^ is attributed to ν_3_ (anti-symmetric stretch) of PO_4_^3−^ ions^39^,^40^,^41^. In addition, the band at 1632.1 cm^−1^ is ascribed to the absorbed water molecule^34^,^35^,^42^,^43^. Lastly, the vibrations of OH^−^ appeared at 3434.8 cm^−1^ 35,43,44,45 in case of x = 0.0. The characteristic FTIR bands are listed in (Table 2).

The splitted weak bands 400–525 cm^−1^ could be assigned to the zirconia; the bands at 427.5 cm^−1^ at x = 0.2 and 525.1 cm^−1^ at x = 1.0 could be attributed to Zr–O-Zr stretching mode^28^,^36^,^37^. Considerably, the small amount of doped zirconia, makes it difficult to differentiate between the bands attributed to HAP and ZrO_2_ as observed from FTIR spectra. It is clear that the band splitting increased with increasing x due to the bands of zirconia that appears as matched with XRD data.

### TGA

**Figure 7:**
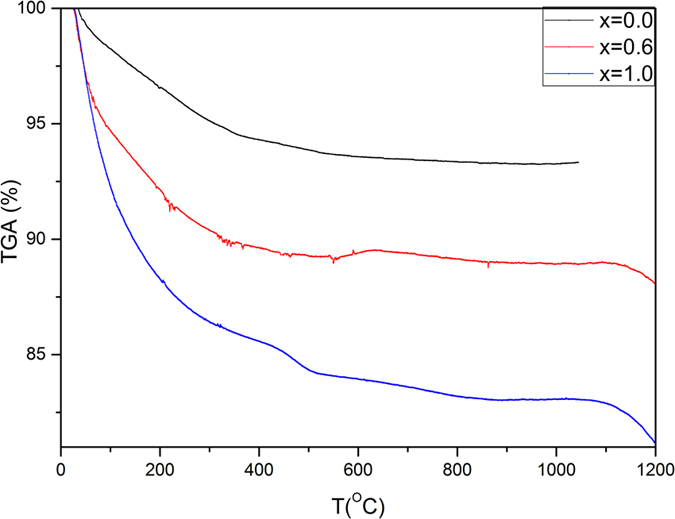
Thermo-gravimetric analysis of Zr-HAP samples at x = 0.0, 0.6 and 1.0.

Thermo-gravimetric analysis (TGA) was carried out for the samples of x = 0.0, 0.6 and 1.0 from room temperature (R. T.) up to 1200 °C as shown in Fig. 7. There is a significant weight loss in all samples; however, the rate of weight loss increases as x increases. The thermograms could be divided into three stages; the first from R. T. up to 200 °C, the second is from 200 to 1000 °C, the third is at T > 1000 °C. The maximum loss at x = 0 doesn’t exceed 6.6% at 1000 °C, while it reaches to 10.7% at the same temperature for x = 0.6. However, it increases significantly from 1000–1200 °C at x = 0.6 to achieve 11.5%. The sample at x = 1.0 showed the largest weight loss which raised to 16.5% at the semi-stable stage up to 1000 °C, and then 18.4% at 1200 °C.

### FESEM

**Figure 8:**
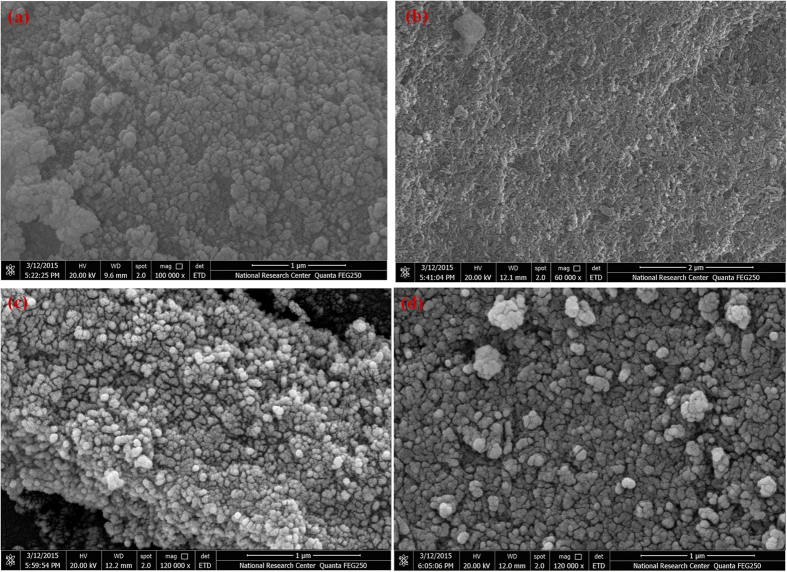
FESEM micrographs of the as synthesized samples at different contents of zirconia; (**a**) x = 0.0, (**b**) x = 0.4, (**c**) x = 0.6, (**d**) x = 1.0.

Figure 8(a) shows the morphology of the as-synthesized Zr-HAP at x = 0.0. This micrograph shows agglomerated hexagonal shape with narrow range distribution around 100 nm. The sample of x = 0.4 at Fig. 8(b); looks as agglomerated rice shape. The surface looks like waves with a little amount of porosity. The rice like particles seem to have a similar size of 130 nm.

Figure 8(c) illustrates the micrograph of the sample with x = 0.6 that looks like highly agglomerated spheres with diameter ranging around 50 nm. The specimen with x = 1.0 is shown at Fig. 8(d), where it appears like cracked granules with size 90 nm.

**Figure 9:**
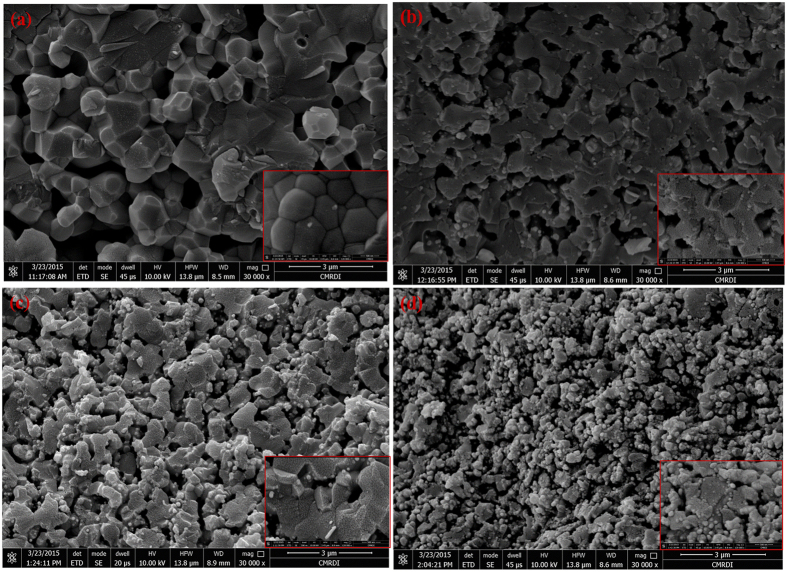
(**a**–**d**) FESEM micrographs of the samples annealed at 1200 °C at different contents of zirconia; (**a**) x = 0.0, (**b**) x = 0.4, (**c**) x = 0.6, (**d**) x = 1.0.

In case of the samples annealed at 1200 °C; x = 0.0 looks like hexagonal crystals with attached boundaries, hard edges and inter-granular porosity. As seen in the micrograph; Fig. 9(a); the surface roughness is very clear at the inset micrograph. The grains have a broad range distribution; however, the average is ∼1.4 μm.

When comparing the sample with x = 0.4 at 1200 °C shown at Fig. 9(b) with that of x = 0.0, the micrograph reveals arranged grain growth with diffused shape and unclear boundaries. The grain size is close to 1.2 μm. The porous nature of the sample is highly pronounced. The nature and type of pores are likely to be closed rather than open ones. The porosity here is specified as an inter-granular type. Furthermore, there is a little amount of small spherical shapes dispersed randomly on the grain boundaries. The small spheres refer to the accompanying secondary phase attributed to zirconia. The later possesses size in the range of 50 nm. The common view of the sample is bone like morphology which is an amazing result.

The predominant shape for x = 0.6 is a hexagonal one while small grains of ZrO_2_ decorating the surface in a more regular pattern. There is a higher amount of porosity than that observed for x = 0.4. However; the pores of x = 0.6 are well distributed and smaller than those at x = 0.4. The pore size is about 0.7 μm. The spherical particles of zirconia tend to fill these open voids as illustrated in Fig. 9(c). Zirconia particles were found to have narrow size about 200 nm.

The last micrograph of x = 1.0 is represented in Fig. 9(d). In this sample; zirconia phase predominates as obtained from XRD. In our case, the microcrack propagation increased in the grain itself and the remaining hexagonal grains of HAP are seemed to be decorated with zirconia nano crystals. HAP grains looks disconnected from each other.

**Figure 10:**
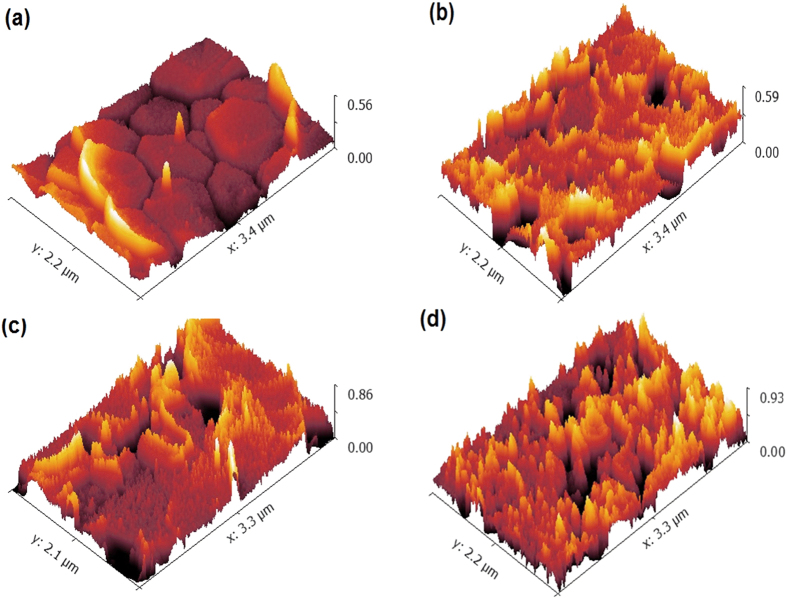
Roughness of samples annealed at 1200 °C; (**a**) x = 0.0, (**b**) x = 0.4, (**c**) x = 0.6 and (**d**) x = 1.0.

The micrographs of FESEM were processed by gwyddion 2.45 software to investigate the surface roughness of the samples, annealed at 1200 °C. Figure 10(a–d) shows the dependence of surface roughness on zirconia content. The mircographs elucidated that the roughness arises as a function of x. (Table 3) shows that the maximum height of the roughness (R_t_) increased from 0.75 μm at x = 0.0 to become 0.83 μm at x = 1.0. The root mean square roughness (R_q_) increased from 0.185 to 0.189 μm from x = 0.0 to 1.0.

### Mechanical properties

The ratio between the negative values of lateral strain and longitudinal strain of a sample is known as Poisson’s ratio^46^,^47^. It could be expressed directly in terms of Miller indices of the lattice planes (hkl) as follows^46^,^48^:


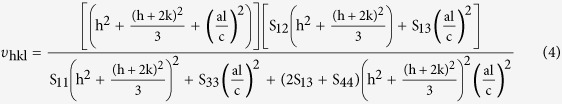


The calculated values of Poisson’s ratio for the annealed samples are reported in (Table 4) where υ decreased in the range of 0.4 < x < 0.6 and then increased again.

The microhardness is defined to be the resistance of materials to be indented^49^. The theoretical microhardness as a function of Poisson’s ratio is extracted from:


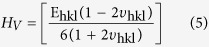


Hardness measurements correlated between the microstructure and mechanical characteristics of a material. The Vickers hardness number (H_V_) is estimated according^50^:


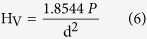


where P is the test applied force, d is the mean length of the indentation diagonals. The experimental test was performed with a load of 100 g for 15 s.

**Figure 11:**
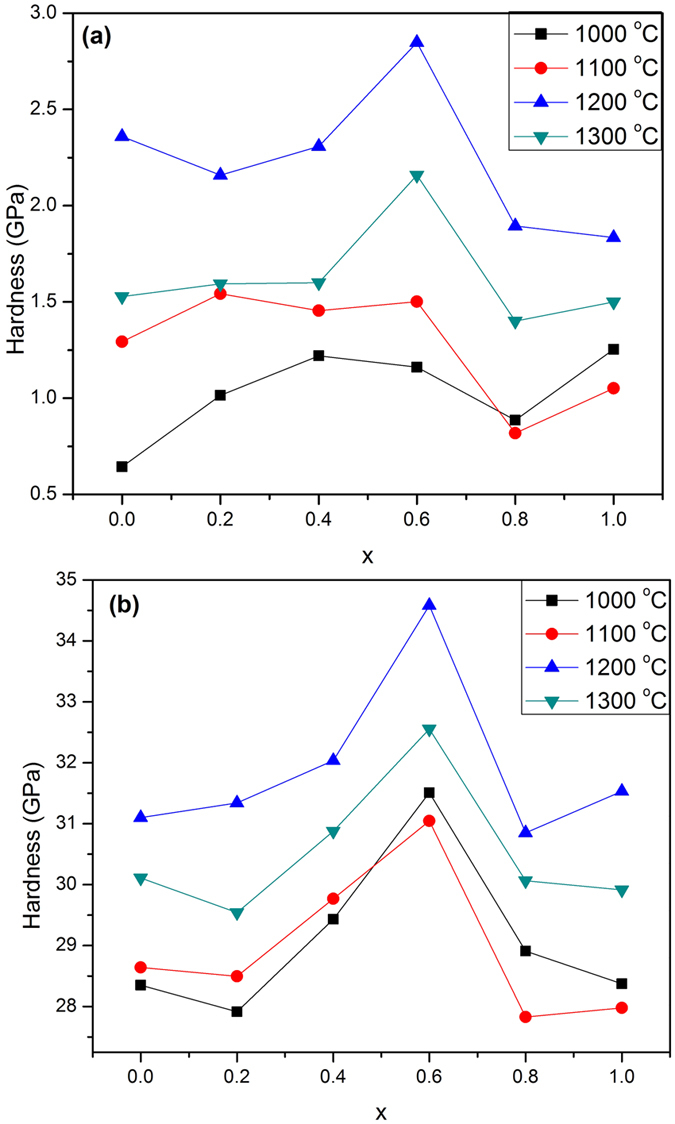
Hardness values of the annealed samples; (**a**) measured hardness, (**b**) theoretical hardness.

Figure 11(a,b) shows the values of the theoretical and measured hardness as a function of zirconia content at different annealing temperatures. From a common view; they have the same trend. The measured hardness increases with increasing x up to the peak at x = 0.6 in all samples, and then decreases significantly. The maximum value is found at 1200 °C and it doesn’t exceed 2.85 GPa. With respect to annealing temperatures; the hardness values were improved with increasing the annealing temperature up to 1200 °C, then dropped down considerably.

In a case of the theoretical microhardness; it is clear that there is a huge difference between theoretical and measured values which reached 20 times. The maximum value of H_v_ was (34.58 GPa) is obtained at x = 0.6, 1200 °C.

**Figure 12:**
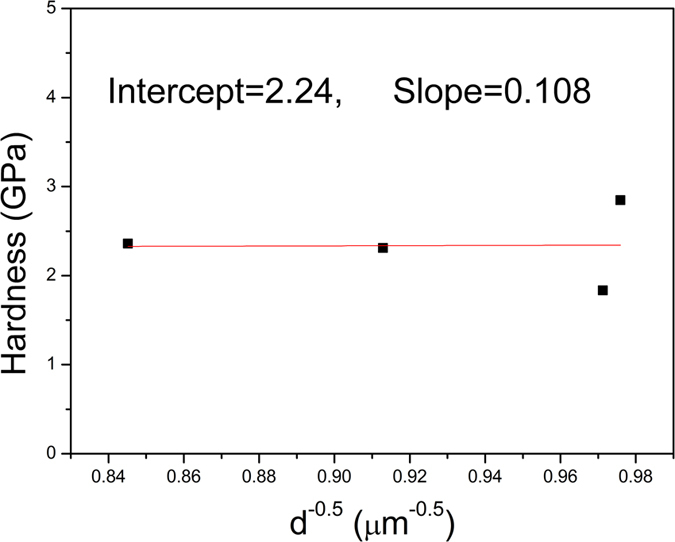
The dependence of measured hardness on the grain size obeying Hall – Petch relation.

It has been well-established that a decrease in grain size results in an increase in the hardness or the yield strength according to the Hall-Petch equation^51^,^52^:


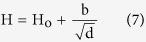


where H represents the hardness and H_o_ represents the hardness of a single crystal of the material under investigation, b is a constant and d is the grain size. The calculated values are illustrated in Fig. 12. Indeed, this relation is obeyed when the grain size is in the range of 1–50 μm.

The bulk modulus - or compressibility (k) - is defined as the hydrostatic pressure necessary to cause a unit relative change in volume of a substance, and it is always a positive value. It could be calculated for hexagonal systems theoretically from^53^:


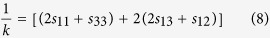


For all samples, it has nearly similar value of 84.6 GPa owing to the hexagonal symmetry.

### Corrosion stability in SBF

**Figure 13:**
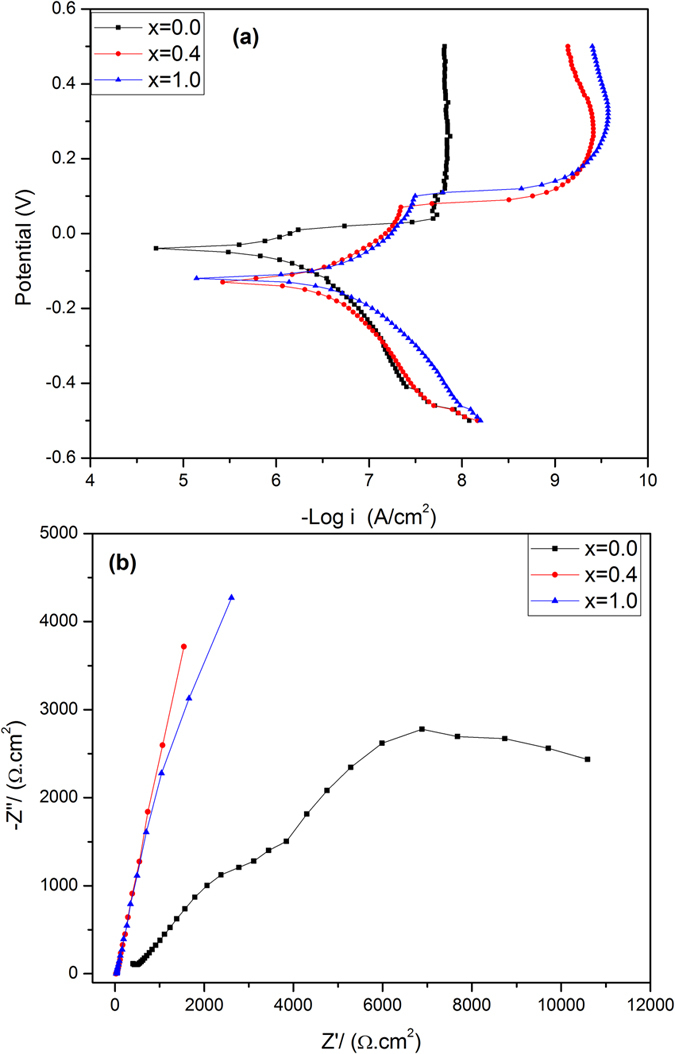
Potentiodynamic polarization curves (**a**) Tafel plot of x = 0.0, 0.4 and 1.0, (**b**) Nyquist plot for the samples x = 0.0, 0.4 and 1.0; immersed in SBF at 37 °C.

The evaluation of the corrosion behavior for the investigated samples annealed at 1200 °C was carried out after immersion in (simulated body fluid) SBF solution at 37 ± 0.5 °C for 24 hrs. Figure 13(a) shows the potentiodynamic polarization curves of Zr-HAP at x = 0.0, 0.4 and 1.0. It is obvious that the corrosion potential (E_Corr_) of the doped samples is shifted to positive direction with a significant decrease in the anodic current. (Table 5) denotes the electrochemical characteristics of the samples. The corrosion current density (I_Corr_) of the samples was significantly decreased with increasing x. The value of I_Corr_ is for x = 1.0 is 12 times lower than that for the pure HAP sample (x = 0.0). Correspondingly, the corrosion rate decreased from 66.76 μm/year for x = 0.0 to 2.589 μm/year at x = 1.0. In addition, the polarization resistance (R_p_) increases significantly with increasing x.

**Figure 14:**
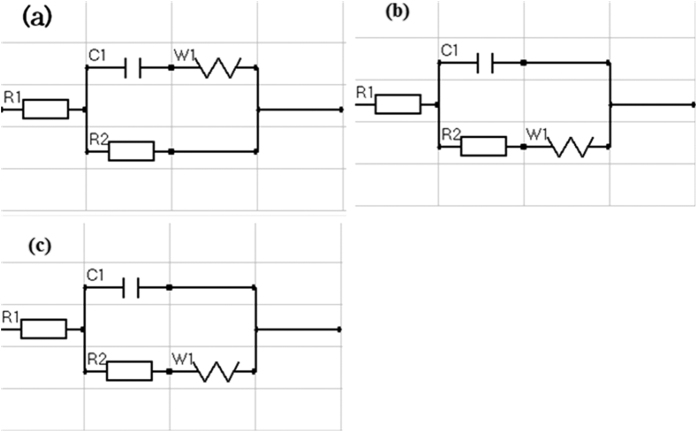
Equivalent electrical circuits (EES) of (**a**) x = 0.0, (**b**) x = 0.4 and (**c**) x = 1.0.

In order to understand the corrosion behavior deeply, electrochemical impedance spectroscopy (EIS) measurements were carried out and illustrated in Fig. 13(b) at x = 0.0, 0.4 and 1.0. The shape of the impedance spectra defines the type of electrochemical reactions that occur on the electrode surface. It is clear that the arc expands with increasing x where the sample at x = 1.0 shows a larger curvature radius in comparison with the others. The EIS data of Zr-HAP samples could be correspondingly fitted with different equivalent electrical circuit (EEC) as clarified in Fig. 14(a–c). EEC is [R_1_ + R_2_(W_1_ + C_1_)] which consists of Ohmic resistance (R_1_) in series with the other part of a circuit and represents the solution resistance, R_2_ in paralleled with the capacitor C_1_. There is a Warburg element (W_1_) in series with the capacitor at x = 0.0 however, it becomes in series with R_2_ at x = 0.4 and 1.0. The equivalent circuit parameters are reported in (Table 6).

**Figure 15:**
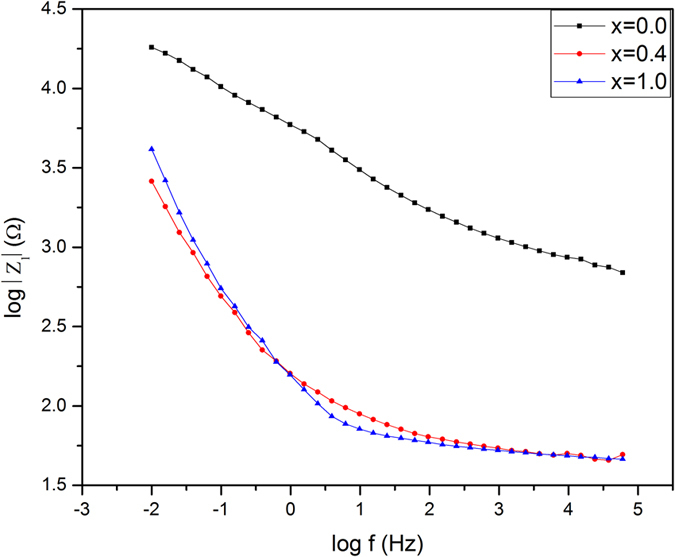
Bode plots of EIS data of samples x = 0.0, 0.4 and 1.0 in SBF at 37 °C.

Figure 15 shows the Bode plots for the same samples. It clarified that x = 0.0 exhibits the highest impedance values, although it reveals the minimum imaginary resistance as illustrated in Nyquist plots.

### Electrical properties

**Figure 16:**
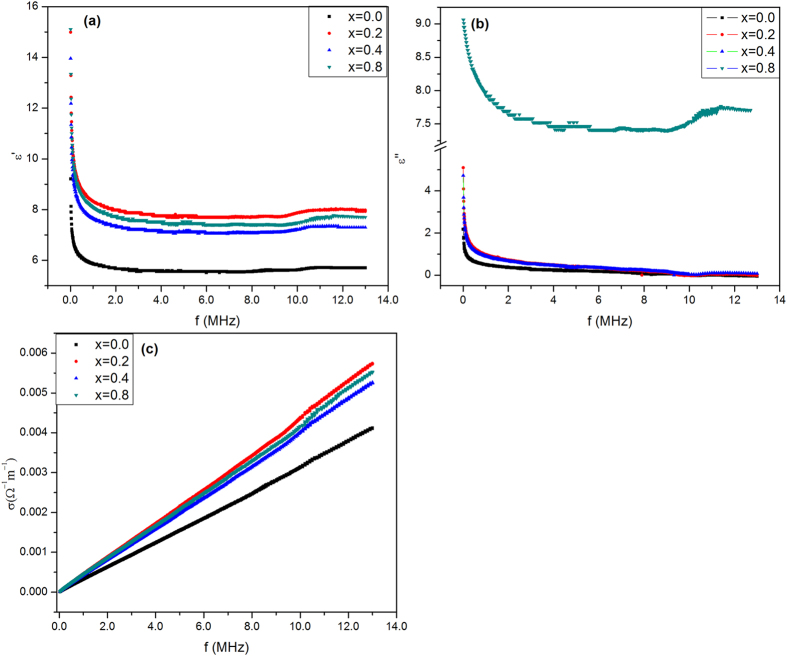
Frequency dependence of (**a**) room temperature real part (ε′), (**b**) imaginary part (ε″) and (**c**) ac conductivity for the investigates samples.

The dielectric constant (ε′) was determined from the measured capacitance of the samples^54^. Figure 16(a) illustrates the variation of ε′ with frequency, where it decreases exponentially with increasing frequency (f) up to 0.25 MHz then becomes nearly stable. Figure 16(b) shows the dependence of the dielectric loss factor (ε″) on the frequency and it reveals the similar behavior of (ε′) except at x = 0.8. The ac conductivity for the investigated samples increases linearly with increasing f as shown in Fig. 16(c).

## Discussion

The detailed phase evaluation using XRD analysis indicates the absence of diffraction peaks corresponding to zirconia phase in the as-synthesized samples. This suggests that zirconia particles were completely entrapped in the HAP lattice. The as-synthesized patterns also exhibit broad diffraction peaks, which is elucidated by poor crystallinity accompanied by small crystallite size.

In the annealed samples; zirconia reveals clear peaks at x ≥ 0.4 (ICDD card no. 01-081-1544), which gives an idea about its solubility limit in HAP matrix. Zirconia secondary phase was crystallized in tetragonal crystal symmetry with space group (P4_2_/nmc).

There is no evidence for any phase transformation of HAP to calcium oxides; α or β-TCP in the samples annealed ≤1200 °C which indicates the high thermal stability of the prepared samples.

Moreover, at higher annealing temperatures i.e T > 1200 °C; HAP decomposed in the presence of ZrO_2_ as follows^25^:


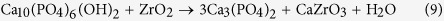


where HAP decomposes into α/β -TCP with the appearance of the second phase of CaZrO_3_. Thus, the decomposition rate of HAP to α/β -TCP is improved with increasing the ZrO_2_ concentration in the samples (zirconia works as a catalyst). Herein, the peak intensities of α-TCP arise with increasing zirconia content. One could argue that the decomposition of HAP–ZrO_2_ started at annealing temperature >1200 °C.

It is noticed that the formed zirconia phase corresponds to ZrO_1.95_ which points to the existence of oxygen vacancies in zirconia unit cell. The so-called polycrystalline tetragonal zirconia displays excellent mechanical properties with an enhanced ionic conductivity at low temperatures as compared to cubic zirconia^55^.

The broad lines of XRD patterns for the as-synthesized samples originated from two sources, namely the small crystal size and the lattice distortion that increased with the incorporation of zirconia into the HAP matrix^56^,^57^. Therefore, the crystallinity is expected to decrease with increasing zirconia content in the sample; as clarified in Fig. 2(a). The diffraction peaks of the annealed samples at Fig. 1(b–e) were sharp and well separated elucidating that the crystallinity, as well as crystallite size, were boosted.

At higher annealing temperatures as well as large zirconia content, the situation is reversed; zirconia becomes the main crystalline phase while HAP appeared a secondary phase at x = 1.0 as in Fig. 1(b–e). Therefore, D_s_ is expected to decrease with increasing zirconia content. This was explained by supposing that zirconia particles acted as a separator between HAP grains owing to existence on the grain boundaries of the later as observed in FESEM. In addition, the difference in the thermal expansion coefficient between hexagonal HAP and tetragonal zirconia crystals could inhibit the lattice expansion and/or crystal growth of HAP grains. The improvement of crystal growth of zirconia on the expense of HAP ones is reported in (Table 1) where D_s_ increased more than 5 times from 1000 to 1300 °C.

The crystallite size computed from W-H method by UDM is usually smaller than its conjugate from Scherrer’s equation because the former takes into consideration the lattice distortion. The most general causes of lattice distortion are dislocations, grain boundaries, microstresses and stacking faults^56^. It was shown that the lattice strain arises with decreasing the crystal size, that is why the lattice strain illustrates opposite trend of the crystallite size.

The lattice distortion (for the as-synthesized samples) is relatively high due to the expected large surface/volume ratio values as a direct consequence of small crystallite size. For the annealed samples, the lattice strain values increased slightly with rising zirconia content. However, microstrains for the as-synthesized and 1000 °C annealed samples, possess high values amongst all since zirconia are incorporated in the HAP crystals inducing more internal strains in the lattice.

The obtained negative values of the microstrains are expected due to the existence of zirconia as a secondary phase on the grain boundaries thereby exerting a pressure on the HAP hexagonal matrix. The external compression could induce the negative strain distortion. However, the positive strain could be attributed to the internal stress in the crystal itself due to the thermal expansion which accompanies the crystal growth^30^. With increasing x, the stress due to zirconia crystals increased. Therefore, lattice strain varies from a negative to a positive value strongly depending on zirconia content. On the other hand, zirconia particles possess a decreasing lattice distortion due to increasing their crystallite size with increasing the annealing temperature.

In performing both USDM and UEDM computations, the anisotropic nature of the elastic constants was taken into consideration whereas the deformation energy density (u) is supposed to have the same value in all crystallographic directions (isotropic nature)^58^. Generally, polycrystalline materials display an anisotropic microstructure^59^. W–H analysis by the three models give more accurate values of crystallite size compared to that computed from Scherrer’s method^58^. Therefore, the crystallite size of USDM and UEDM show similar trend which is inversely proportional to the stress (σ). The latter produced by zirconia particles on HAP crystals acts as a dispersing factor on HAP lattice, that is why the stress is increased significantly with increasing x. The crystallite size and the lattice strain values estimated from USDM and UEDM models could be further extended to calculate the dislocation densities present in the samples with more accuracy^58^.

Pure hydroxyapatite unit cell is described in terms of a hexagonal structure with dimensions a = b = 9.432 Å, c = 6.881 Å, α = β = 90°, γ = 120° adopting the space group P6_3_/m^60^. The main structural evidence reported in Fig. 4(a,b) was the decrease of (a) and the increase of (c) parameters, together with the absence of secondary phases and the increase of tetrahedral distortion^61^.

In case of the as-synthesized samples, the decrease of (a) could be explained by supposing that ions with smaller size than Ca^2+^ or phosphate ions have occupied some position in HAP unit cell and one assumed four probabilities: The 1^st^ is that Zr^4+^ could replace Ca(I) ion positions, 2^nd^: Zr^4+^ replace P^5+^ positions, 3^rd^: ZrO_1.95_ substitute the phosphate group and the last one is that carbonate substitutes the phosphate position which is categorized as (B-type). The ionic radii of Zr^4+^ is larger than that of P^5+^, therefore, the 2^nd^ probability is not preferred^14^. B-type of carbonated substitution (4^th^) is evident from FTIR spectra. In addition, there is no significant A type. However, the decrease of (a) constant stopped at x = 0.4, then rise up to x = 1.0. This increase of (a) may due to an incorporation of ZrO^2+^ into Ca(I) position. ZrO^2+^ has a radius (2.1 Å) which is more than 2 times of that of Ca^2+^ radius (0.99 Å)^25^,^62^. The augmentation of (c) constant with increasing x may be explained by assuming the rearrangement of ZrO^2+^ or ZrO_1.95_ in c- direction.

For the annealed samples, one could expect the redistribution of ZrO^2+^ among the available Ca^2+^ sites. Moreover, it was believed that in some cases, high temperature site preference plays a significant role in the appearance of tetragonal zirconia phase especially at large zirconia contents. Therefore, the lattice parameters in case of the annealed samples represent opposite trends where (a) constant has higher values at x = 0.4 than the other zirconia contents. This is directly related to its solubility limit in the HAP matrix. Above x = 0.4, zirconia could exist as a separate phase as matched from XRD results. The (c) constant keeps the same trend of the as-synthesized samples because it is related to the crystallographic direction of the substituting positions.

The (c/a) ratio is close to the trend of (a) more than (c) because the value of (a) is larger than (c). One demonstrated that (c/a) values decay with increasing the annealing temperature owing to the inhibition of crystal growth in (c) direction. This will induce the formation of more spherical shape (as matched with FESEM at Fig. 9(d)). Accordingly, the anisotropy will be reduced^31^,^63^. Consequently, the lattice strain is expected to decrease with increasing the annealing temperature, which agreed well with lattice strain results as shown in Fig. 3(a).

TGA analysis could be explained by supposing three steps of weight loss. In the first stage; the weight loss is associated with the water and residual solvent removal, which comes from the aqueous medium. It is recognized by high accelerating rate of weight loss. Moreover, the weight loss rate increases with x, which could be attributed to lowering crystallinity. Consequently, at 100 °C, x = 1.0 loses 7.6% while x = 0 loses 1.7% only. The second stage is up to 1000 °C, which show clear plateau due to the distinguished high thermal stability of zirconia/HAP composite. The third step is accompanied by structural phase transition of zirconia from monoclinic to tetragonal phase.

General view of the FESEM micrographs of the as-synthesized samples clarifies that there are no traces of other materials which matched with the results explained in XRD. Moreover, it proves that zirconia has been integrated into HAP crystals assuming a volume diffusion mechanism. In addition, the porosity is classified as inter-granular type, where the crack propagates along the grain boundaries. This may be attributed to the high ratio of interconnected porosity. This type of brittleness at the grain boundaries is generally a distinguished sign of ceramics^31^. The grains are interconnected together, which look as they are fused grains. Accordingly, there is a considerable ratio of trans-granular porosity in the range of 0.9 μm which is classified as macro porous^64^.

It is noted that, with increasing dopant concentration in the samples, the grains tend to crumble. However, zirconia particles are oriented to fill the pores, hence they works as a cement for HAP grains or could be named as network filler. The grains of zirconia grow from 50 nm at x = 0.4 to 200 nm at x = 0.6, which give them the chance to interconnect HAP grains. Therefore, it is expected to enhance the mechanical properties up to a saturation limit. When exceeding this limit, HAP grains look as disintegrated particles as shown in x = 1.0 at 1200 °C (Fig. 9(d)). The grain boundaries and interfaces have a different structure from the bulk grains. The former reveals high catalytic and surface activity than the bulk. That is why zirconia particles tend to agglomerate on the grain boundaries. The situation is the lattice mismatch between the hexagonal crystal of HAP and tetragonal one of ZrO_1.95_. Zirconia is thought to diffuse out of the grain boundaries by a pining diffusion mechanism rather than a volume diffusion one.

Increasing zirconia content to x = 0.6, supports our hypotheses about surface pining diffusion, especially the pores are interconnected and they are open pores as illustrated in Fig. 9(c). Additionally, the existence of some microcracks at the grain boundaries is a direct consequence of the lattice mismatch as well as the difference in thermal expansion coefficient of both crystalline phases, namely hexagonal (HAP) and tetragonal (ZrO_1.95_). This behavior is reflected in the hardness values.

The spherical shape (at x = 1.0) represent zirconia particles with size 135 nm. In addition, there is a large amount of disconnected pores with size about 1.4 μm. The overall argument is that the existence of zirconia secondary phase in the HAP at this annealing temperature (1200 °C) enhanced the densification in a limited proportion while the porosity is less pronounced. This could be highly employed in biomedical implants where the surface roughness along with porous nature of the bioceramics is highly desirable. In addition, the grain size decreased from 1.4 μm at x = 0.0 to 1.06 μm at x = 1.0. This decrease is mainly attributed to the strain caused by zirconia phase on the grain boundaries of the hexagonal one.

The biological HAP doesn’t possess a smooth surface because of a large number of structural substitutions^65^. The surface morphology of the synthsized HAP depends not only on the substitution ratio but, also on the preparation conditions, like pH value. However, with increasing the pH value, the crystal growth tends to be more isotropic or weak anisotropic^63^. Therefore, at constant pH value, the substitution content becomes the crucial factor affecting on the surface morphology. The extreme roughness including the surface irregularities in the nano regime, theoretically coincides with the tendency to increase the binding of biological molecules which improve the biocompatibility and osteointegration. In addition, it has a positive influence on the inflammatory reactions^66^. Therefore, recent studies tend to imitate those natural rough HAP. Moreover, the increase in surface roughness allows to enlarge the contact part with the host tissue as well as increasing the integration paths and consequently develop the mechanical interlocking between the implant and the host tissue which improve the adhesion/tension force between them. According to recent studies, it was also concluded that the surface roughness is extremely important in facilitating the implantation of bone integration^66^,^67^.

The computed Poisson’s ratio takes into consideration the anisotropic nature. All annealed samples could be considered as a partially auxetic materials. The later is a type of materials that exhibit a negative Poisson’s ratio where it expands under the applied stress^68^. It is demonstrated that the concentration of x = 0.4 displays higher 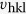 than that of other samples which leads to high indentation resistance. This expectation is matched well with the hardness results.

The hardness is governed by the grain size, porosity, densification and/or existence of a secondary phase. Figure 11(a,b) illustrates the dependence of the measured and theoretical microhardness on zirconia content. The difference between them originated from (i) the porosity which is not taken into consideration in the equation of (E_hkl_). (ii) The microcracks formed in the ceramic samples which are observed in FESEM micrographs. (iii) The existence of a secondary crystalline phase (ZrO_1.95_) which has different hardness values and affected strongly on the overall values. The increase in hardness with increasing zirconia content is mainly due to the decrease in grain size. For the samples annealed at temperatures higher than 1000 °C, the hardness increases up to x = 0.6 and then decreases, where the maximum hardness is achieved at x = 0.6. Hardness values are obviously rising with the annealing temperature due to enhanced densification and compactness. However, the hardness decreased at T > 1200 °C because of crumbled grains due to the significant ratio of zirconia on the grain boundaries, the phase transition of HAP to α-TCP is a factor working on reducing the hardness to 1.4 GPa. The measured values of the hardness are in good correlation with the appearance of zirconia phase. The porosity is lowered with zirconia content up to x = 0.4 and then increased. This finding is in line with the hardness as the later gives the opposite trend. The samples annealed at 1200 °C didn’t follow the same trend as the value of porosity is lower than that obtained for corresponding annealing temperatures. Minimum porosity at x = 0.6 at 1200 °C coincides with the maximum hardness. With decreasing the grain size, the relative volume of grain boundaries increases, leading to an increase in the resistance to indentation. Therefore, the presence of zirconia inhibited the grain growth of HAP particles. Thus, the annealed samples containing zirconia nano spheres can be produced by further consolidation, for its developed strength^25^. On the other hand, the lattice distortion on the surface or at the interface between grains could strongly affect on the surface or interface free energy and consequently change effectively the mechanical properties^30^.

Moreover, it was found that the samples obeyed Hall–Petch relationship, where the hardness decreased with increasing grain size. This phenomenon is displayed from the dislocation movement or migration. The later could be inhibited by grain boundaries; therefore by decreasing the grain size, the dislocation free path will be consequently reduced^69^.

The computed bulk modulus displays one value because it doesn’t depend on the lattice parameters. W.Y. Ching *et al*. calculated the bulk modulus^67^,^70^ of HAP and it was 84.51 GPa while it was 89.0 with N. Y. Mostafa which is very close to our results.

The increase of polarization resistance (R_p_) with increasing the zirconia content indicates an improvement in the corrosion resistance^71^. This inhibition of electrochemical activity by increasing x could be attributed to the novel role of zirconia in the aqueous solution. In addition, the decrease of the grain size with increasing zirconia content could delay the reaction with the surface particles. The sample of x = 1.0 revealed largest EIS semicircle relative to the other ones which points to a remarkable improvement of the sample corrosion resistance. The large impedance values at x = 1.0 relative to the other samples implies that diffusion of ions out of the sample to the electrolyte, is inhibited subsequently minimizing the corrosion rate. The corrosion rate was found to decrease 23% for the sample at x = 0.4 with respect to the pure one. It is clear that the characterized EIS are corroborative with the results obtained using Tafel study.

The centers of Nyquist semicircles seem to be situated below the Z′ axis, meaning that the samples contain inhomogeneity. The later was formed during the annealing process at elevated temperatures and are evident from the oxygen deficient zirconia phase (ZrO_1.95_) as identified from XRD results. Another possible reason is the appeared porosity (calculated porosity values) as in line with FESEM observations.

The sample of x = 0.0 reveals two semicircles pointing to the existence of two different relaxation times. The first one is associated with the grains (HAP) and the 2^nd^ is attributed to grain boundaries. Furthermore, with x = 0.0, the W_1_ element was found to be in parallel with R_2_ while it is fitted to exist in series with R_2_ for x = 0.4 and 1.0.

The large capacitance values as obtained from the Nyquist plot fitting pointed to large relaxation times. This could be advantageous for several applications. However, in Bode plots, the pure HAP at x = 0.0 displays the highest total impedance. Therefore, the Ohmic resistance is the main contributor to resistance in this case and could be explained by supposing that zirconia particles improved the conductivity. Further studies are needed at high temperature to assure the evidence of ionic conductivity in the doped samples.

The large values of (ε′) at lower frequencies are attributed to space charge polarization and the alignment of dipoles in the direction of the field. This enhances the obtained large relaxation time values for (Z′–Z″) plots as mentioned above. As the frequency increases the electron exchange cannot follow up the electric field variation and the polarization becomes frequency independent as shown in Fig. 16(a).

The similarity between the dielectric constant and dielectric loss behaviors; (except at x = 0.8); could be clarified by increasing the ratio of zirconia which is distinguished by oxygen vacancies. The later acts as a trapping center for the charge carriers which lead to greater loss values. The ac conductivity (σ_ac_) was calculated and presented as a function of frequency in Fig. 16(c). The increase in frequency resulted in the increase in conductivity where the former acts as a pumping force for the charge carriers’ movement^72^. It is clear that (σ_ac_) of the doped samples is higher than that of the pure sample at the same frequency, due to the existence of holes owing to the oxygen vacancies in zirconia crystal (ZrO_1.95_). The development of the polarization in the samples could improve the bone growth^73^.

## Methods

All raw materials used in the preparation were analytical grade reagents and used as received without any further purification. Hydroxyapatite doped with zirconia [Ca_10_(PO_4_)_6−x_(ZrO_2_)_x_(OH)_2_]; (0 ≤ x ≤ 1) in step 0.2 was synthesized using Co-precipitation assisted microwave irradiation. The ratio of Ca/(Zr + P) was adjusted to be 1.67 for all concentrations as the equation:


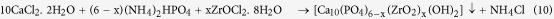


**Figure 17:**
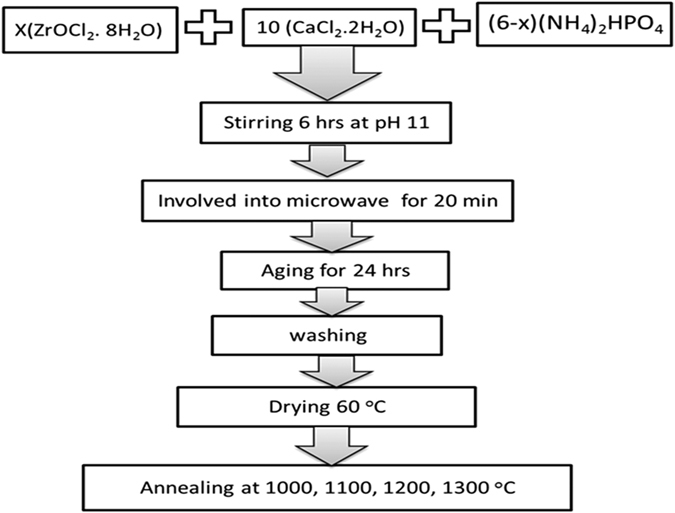
Block diagram of preparation method.

The aqueous solutions of [(NH_4_)_2_HPO_4_] and zirconium oxychloride [ZrOCl_2_. 8H_2_O] were slowly dropped on CaCl_2_. 2H_2_O with continuous stirring. The pH of the solution was adjusted and kept at 11** ± **0.05 for all concentrations. The stirring was maintained at a constant speed for 6 hrs. The obtained suspensions were involved into the microwave in a water bath for 20 mins as follow: 5 mins ON, 4 mins OFF. The solutions were aged for 24 hrs to precipitate, then thoroughly washed with doubled distilled water and dried at 60 °C. The obtained powders were pressed into pellets with 4 mm radius, using a uniaxial press of 60 MPa for 10 sec. The pellets were annealed in Lenton furnace (UAF16**/**5) at different temperatures at; 1000, 1100, 1200 and 1300 °C for 1 h in air with a heating/cooling rate of 10 °C/min. The block diagram illustrating the synthesis procedure is shown in Fig. 17.

X-ray diffraction analyses were carried out using (analytical-x’ pertpro with Cuk_α1_ target, λ = 1.5404 Å, 45 kV, 40 mA, Netherland) to identify the formation of the samples in the desired phases. All the diffraction charts obtained were scanned in the range 4 ≤ 2θ ≤ 60° of angles with step size of 0.02 and a step time of 0.5 s. The corrected broadening β_hkl_ equivalent to the diffraction peak of HAP was estimated using the equation^58^:


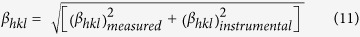


The crystallinity is represented by the fraction of crystalline phase available in the analyzed samples and calculated from the equation^74^:


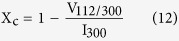


where X_c_ is the degree of crystallinity, I_300_ is the intensity of the (300) diffraction peak and V_112/300_ is the hollow intensity between (112) and (300) peaks of HAP, respectively.

The crystallite size D_s_ was computed from Scherrer’s equation^7^:


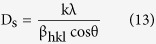


where D_s_ is the Sherrer’s crystallite size (nm); k is the shape factor (k = 0.9), λ is the target wave length of the X-ray (λ = 0.154056 nm for CuK_α_ radiation); θ is Bragg diffraction angle (°) and β is the corrected full width at half maximum (in radians).

The microstrain resulting in line broadening β is calculated from the relation^58^:


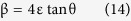


By considering that the present strain is uniform, which reflects the isotropic nature of the crystal, Williamson–Hall (W-H) equation is expressed as^58^:


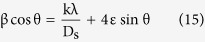


By plotting (β cos θ) on the y-axis and (4 sin θ) on the x-axis, the crystallite size was obtained from the y-intercept and the lattice strain from the slope of the linear fitting.

By supposing small strains to exist in the as-synthesized and annealed samples, the stress-strain behavior will obey Hook’s law as^75^:


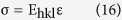


where (E_hkl_) is the modulus of elasticity or Young’s modulus. As a result, Eq. 15 may be modified to represent the USDM where^58^:


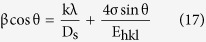


The uniform stress can be obtained from the slope of the straight line plotted between 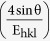 and β cos θ. (E_hkl_) is determined for hexagonal systems from the following relation^48^:


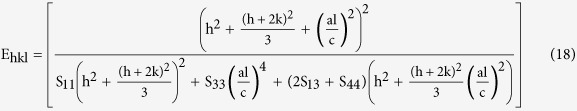


where S_11_, S_13_, S_33_, and S_44_ are the elastic compliances and their values are 7.49 × 10^−12^, 4.0 × 10^−12^, 10.9 × 10^−12^ and 15.1 × 10^−12 ^m^2^/N respectively^58^,^75^.

For a tetragonal polycrystalline zirconia, Young’s modulus is calculated from^76^:


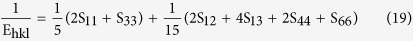


where S_11_ = 3.46 × 10^−12^, S_33_ = 4.06 × 10^−12^, S_12_ = −0.96 × 10^−12^, S_13_ = −0.59 × 10^−12^, S_44_ = 17 × 10^−12^, S_66_ = 15.4 × 10^−12 ^m^2^/N^76^. In addition, the y-intercept represents the crystallite size depending on the USDM system.

The energy density (U: energy per unit volume) could be substituted for 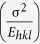 in Eq. 17 to be modified into the form^58^:


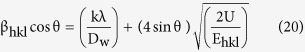


The plot of β_hkl_  cos θ on the y-axis and 
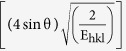
on the x-axis represents UEDM.

FT-IR spectrometer (Perkin-Elmer system 2000) was used for recording FTIR spectra in the range of 4000–400 cm^−1^. Thermo-gravimetric (TGA) analysis was carried out from room temperature up to 1200 °C in a DTG-60 SHIMADZU analyzer using an air flow rate of 100 ml/min and a heating rate of 10 °C/min. The surface morphology was examined using field emission scanning electron microscope (FESEM) model QUANTA-FEG250 (Netherland). Micrographs were recorded at an operating voltage of 10 kV on the powdered samples. Microhardness experiments were carried out using (TTS UNLIMITED INC. model: HWDM-7/Japan) with situ imaging mode. Corrosion behavior of the annealed samples was evaluated in SBF as previously reported by D. Qiu *et al*.^29^. A conventional electrochemical cell with three electrodes was used for corrosion measurements which consisted of standard three electrodes where Pt net as the counter electrode, a reference electrode and the sample is considered as the working electrode. Each sample has a surface area = 1 cm^2^. The temperature of SBF solution was adjusted at 37 ± 0.5 °C. Open circuit potential was working for 1 h to achieve the steady state. For Electrochemical impedance spectroscopic analysis (EIS), a frequency range was applied from 0.01 Hz up to 60 kHz. The potential was applied from −0.5 to +0.5 V. EIS data were analyzed using Ivium software. Dielectric and ac conductivity measurements were carried out at room temperature on a disk shaped sample coated with silver paste using LCR meter model (HP 4192A).

## Conclusion

HAP doped with zirconia nanoparticles were successfully synthesized using microwave assissted co-precipitation method. The annealing temperature was found to play a significant role in the phase formation as well as the crystallinity and the appearance of the secondary phases. The mechanical properties were highly accentuated up to x = 0.6 and then decayed. Additionally, the highest measured hardness value was obtained at x = 0.6 which was annealed at 1200 °C with value 2.85 GPa. The calculated Poisson’s ratio showed a partially auxetic behavior. The hardness was found to satisfy Hall-Petch relation. In addition, a nice correlation was established between measured, calculated physicomechanical properties and the observed morphology. The corrosion resistance increased 1.67 times by increasing zirconia content from x = 0.0 to x = 1.0 which means that zirconia was successful as a corrosion inhibitor. Owing to the superior mechanical properties of the samples annealed at 1200 °C, the discussed results may suggest a new methodology for fabricating biomimetic nano doped HAP. Other potential applications could be suggested upon future investigations.

## Additional Information

**How to cite this article:** Mansour, S. F. *et al*. Physico-mechanical and morphological features of zirconia substituted hydroxyapatite nano crystals. *Sci. Rep.* **7**, 43202; doi: 10.1038/srep43202 (2017).

**Publisher's note:** Springer Nature remains neutral with regard to jurisdictional claims in published maps and institutional affiliations.

## Figures and Tables

**Table 1:** The crystallite size using different models: lattice strain, stress and the energy density of zirconia at x = 1.0 at different annealing temperatures.

T(°C)	D_s_ (nm)	UDM (nm)	ε × 10^−3^	USDM (nm)	UEDM (nm)	Stress (MPa)	U (kJ/m^3^)
1000	11.6	11.2	0.35	11.2	11.2	66.3	11.4
1100	17.4	15.4	−0.39	15.4	15.4	−74.4	14.4
1200	22.4	18.9	−2.78	18.9	18.9	−244.6	155.7
1300	56.6	39.6	−1.37	27.1	27.1	−263.8	181.1

**Table 2:** Characteristic transmittance infrared bands of Zr-HAP with different concentrations of zirconia.

x = 0.0	x = 0.2	x = 0.4	x = 0.6	x = 0.8	x = 1.0	Assignment	References
—	427.5	—	—	—	—	Zr–O stretching mode	28
447.5	—	—	—	—	443.2	ν_2_ bending mode PO_4_^3−^ ions	35,41
—	—	—	—	—	461	Zr-O stretching mode	37
467.9	466.7	468	468.4	468	467.7	ν_2_ Vibration O–P–O	33,38
—	—	—	—	—	525.1	Zr–O stretching mode	28
562.8	563.3	563.4	562.4	563.97	563.3	ν_4_ bending mode of O–P–O	33
602.5	602.9	603	602.8	603.3	602.2	ν_4_ bending mode of O–P–O mode	33,34
874	874.4	874	873.4	873.5	872	ν_2_ of vibrations of CO_3_^2−^	33
1030.3	1031.3	1031.8	1031.5	1031.6	1031.6	ν_3_ anti-symmetric stretch of PO_4_^3−^	34,35,42
1421.8	1422.98	1421.7	1423.2	1423	1421.7	ν_3_ of CO_3_^2−^ ion	33,38
1453.7	—	—	—	—	—	Vibrations of CO_3_^2−^	33,38
1632.1	1642.96	1640.6	1633.5	1634.1	1631.1	Absorbed water	43
3434.8	3434.96	3437.8	3439.4	3434.7	3428.4	The vibrations of OH^−^	35,43,45

**Table 3:** Roughness parameters for the annealed samples of x = 0.0, 0.4, 0.6 and 1.0.

x	R_a_ (μm)	R_t_ (μm)	R_q_ (μm)
0.0	0.173 ± 0.45450	0.7508 ± 0.3567	0.185 ± 0.071040
0.4	0.1292 ± 0.0238	0.7927 ± 0.3209	0.1579 ± 0.05448
0.6	0.1638 ± 0.0360	0.7862 ± 0.3321	0.1918 ± 0.06651
1.0	0.1567 ± 0.2890	0.8321 ± 0.3067	0.1893 ± 0.05745

**Table 4:**
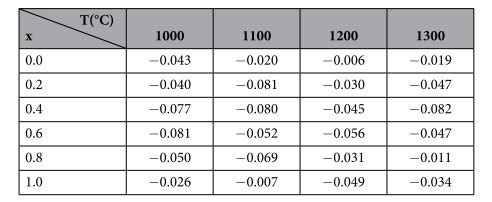
The calculated Poisson’s ratio for all annealed samples.

**Table 5:** The corrosion parameters determined from the Tafel plot for the samples at x = 0.0, 0.4 and 1.0.

x	E_Corr_ (V)	I_corr_ (μA/cm^2^)	β_a_ (V/decade)	β_c_ (V/decade)	C. rate mm/y	R_p_ (Ω)
0.0	−0.5795	62.4	19.548	0.432	0.06676	5041
0.4	-0.4360	20.0	0.293	0.346	0.01601	5805
1.0	−0.1305	5.211	0.154	0.296	0.00259	8430

**Table 6:**
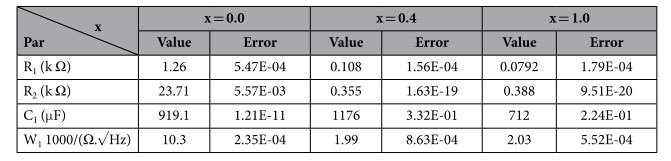
Equivalent circuits’ parameters of the samples at x = 0.0, 0.4 and 1.0.
